# Editorial: New ideas and international perspectives on school bullying: a multidisciplinary approach

**DOI:** 10.3389/fsoc.2026.1870043

**Published:** 2026-05-27

**Authors:** Christopher Donoghue, Dorothy L. Espelage, Robert Thornberg

**Affiliations:** 1 Montclair State University, Montclair, NJ, United States; 2 University of North Carolina at Chapel Hill, Chapel Hill, NC, United States; 3 Linköping University, Linköping, Sweden

**Keywords:** bullying, cyberbullying, education, psychology, sociology

In 2022, two of the editors of this Research Topic (Donoghue and Espelage) joined a team of bullying researchers from around the world to establish a new international definition of “school bullying” for UNESCO and the World Anti-Bullying Forum (WABF). The new definition reads as follows:

*School bullying is a damaging social process that is characterized by an imbalance of power driven by social (societal) and institutional norms. It is often repeated and manifests as unwanted interpersonal behaviour among students or school personnel that causes physical, social, and emotional harm to the targeted individuals or groups, and the wider school community* (O'Higgins Norman et al., 2024).

This Frontiers Research Topic explores several dimensions of this definition with a special emphasis on: (a) the changing digital and cultural contexts for aggression; (b) multilevel social ecological explanations; (c) the importance of not ignoring individual-level factors; (d) social and psychological factors that mediate in bullying situations; and (e) preventive strategies and policies that reduce bullying. The result is a series of 15 papers on new ideas and international perspectives on school bullying which offer a view to the future direction of bullying research. In this introduction, we briefly review these works and consider how they connect to the UNESCO definition.

Beginning with the studies on the changing digital and cultural contexts for bullying, Putri et al. used the Netflix series *Adolescence* as a case study to demonstrate the intergenerational disconnect that can form when context-dependent emojis, digital slang, and memes are employed in interactions among young people. The confusing nature of these communication forms can lead to obvious gaps in the ability of people of older generations to understand, react to, and protect them from the harms of bullying. In this work, as well as in Prestera et al., we are reminded of the degree to which bullying is socially constructed and context-specific. In Prestera et al., we further see how actor intentions in cyberaggression may be interpreted as willful, even in situations when actors are following perceived social norms or yielding to peer pressure. Collectively, these studies on digital and cultural contexts suggest it is vital for any researcher or school administrator to take social norms into account when aiming to understand aggressive intentions and victim impact.

A wider angle lens is employed in the next series of papers to investigate the social ecological order in bullying situations. At the macro level, Sun and Zhao considered the effects of anti-bullying laws in the United States and observed that although they have had limited effects on social-behavioral skills, they have been impactful in improving children's interpersonal skills. One explanation for the uneven impact of anti-bullying laws is the differential role of mediating factors at the meso level of the social environment. At the meso level, Ma and Niu found that teachers can enhance their classroom climate and student relationships when they possess a higher level of social-emotional competence, and this may lead to lower bullying prevalence. Multilevel studies like Wang et al.'s demonstrate that all levels (micro, meso and macro) play a role in bullying and point to the ways that school-type can moderate the meso-level factors such as parental discipline. These studies provide evidence for the UNESCO claim that bullying is a damaging social process involving not only the targeted individuals and groups, but also the broader community.

Although the UNESCO definition emphasizes that bullying is a social process driven by social (societal) and institutional norms, it is important not to ignore or overlook other possible causes or factors. Several studies in this Research Topic draw attention to how individual factors are involved in bullying. For example, Hinduja and Patchin found that hope can serve as a motivational and cognitive resource that is protective against bullying and cyberbullying. Siddiqui et al. found that feelings of moral disengagement can serve as a facilitator for bullying, especially as a means for achieving power and superiority. Mizuno's research found that ultimate just-world beliefs can lead to victim-blaming and a lesser chance of recognizing bullying behavior when it is being witnessed. Finally, in Carvalhais and Vargos, adolescents may strategically combine prosocial and aggressive behaviors as adaptive responses to bullying, indicating that there may be more than one pathway to emotional wellness when dealing with aggression.

According to the UNESCO definition, school bullying causes physical, social and emotional harm. Several papers in this volume address that harm and consider the ways that these harms can be mediated by social and/or psychological attitudes. In Wen et al., the presence of alexithymia (or the difficulty in identifying and expressing emotions) predicted whether a victim will experience non-suicidal self-injury (NSSI) as a result of victimization. Lan et al. exemplifies how bullying is associated with harm (depression and academic anxiety), but also how a psychological variable (academic anxiety) mediates the relationship between bullying and harm (depression). Molero Jurado et al. likewise demonstrates the relationship between bullying (cybervictimization) and harm (negative emotional symptoms) and how this relationship is mediated by psychological flexibility.

Finally, in three studies on prevention and intervention strategies, a light is cast on institutional norms referenced in the UNESCO definition. For prevention, Ma et al.'s research found that sports participation can bring benefits such as mental toughness and resilience which are protective against bullying. In another study of an anti-bullying intervention effort, Shao et al. found that young children demonstrated a greater level of motivation to learn about bullying when they were enabled to do so with a gamified interactive e-book. Lastly, Velarde-Camaqui et al.'s review of the history of psychological interventions in school bullying describes the trend away from behavioral approaches toward more systemic institutional strategies. Although this study concurs with previous studies indicating inconsistent results across anti-bullying programs, the authors found that models employing cognitive-behavioral and social-emotional learning models have been met with the greatest success.

Taken together, this Research Topic highlights the value of examining and theorizing social factors and processes to understand bullying, which is consistent with UNESCO's definition. Bullying is undoubtedly a social phenomenon embedded in a multifaceted social context. However, although the UNESCO definition emphasizes that bullying is a social process driven by social (societal) and institutional norms, it is important not to ignore or overlook individual factors (e.g., cognitions, motivations, emotions, social-emotional competence) that research has also shown to be important, as well as other social factors than norms (e.g., parenting, home–school cooperation, sport participation, school type, classmate relationships, friendship quality). The research in this volume supports inclusive theoretical approaches, such as social-ecological, social-cognitive, and sociocultural perspectives, which posit that bullying results from a complex interplay of individual and contextual factors (from micro- to macro-level), and underscores the importance of not reducing our understanding of bullying to a single level of explanation or a simple cause. A continued and deepened dialogue between different disciplines within the bullying research field can contribute to more advanced theoretical syntheses that better help us understand, predict, prevent, and address bullying.
